# Protocol to Isolate a Large Amount of Functional Oligodendrocyte Precursor Cells from the Cerebral Cortex of Adult Mice and Humans

**DOI:** 10.1371/journal.pone.0081620

**Published:** 2013-11-26

**Authors:** Eva María Medina-Rodríguez, Francisco Javier Arenzana, Ana Bribián, Fernando de Castro

**Affiliations:** Grupo de Neurobiología del Desarrollo-GNDe, Hospital Nacional de Parapléjicos, Toledo, Spain; IIBB-CSIC, IDIBAPS, CIBERNED, Spain

## Abstract

During development, oligodendrocytes are generated from oligodendrocyte precursor cells (OPCs), a cell type that is a significant proportion of the total cells (3-8%) in the adult central nervous system (CNS) of both rodents and humans. Adult OPCs are responsible for the spontaneous remyelination that occurs in demyelinating diseases like Multiple Sclerosis (MS) and they constitute an interesting source of cells for regenerative therapy in such conditions. However, there is little data regarding the neurobiology of adult OPCs isolated from mice since an efficient method to isolate them has yet to be established. We have designed a protocol to obtain viable adult OPCs from the cerebral cortex of different mouse strains and we have compared its efficiency with other well-known methods. In addition, we show that this protocol is also useful to isolate functional OPCs from human brain biopsies. Using this method we can isolate primary cortical OPCs in sufficient quantities so as to be able to study their survival, maturation and function, and to facilitate an evaluation of their utility in myelin repair.

## Introduction

Multiple sclerosis (MS) is a severe chronic disease that affects more than 500,000 people in the EU alone and approximately 2,500,000 people worldwide. Indeed, MS represents the most frequent neurological disorder in young adults and it is the second most important cause of paraplegia, among other severe disabilities. To date, the only treatments available to combat MS involve the use of immunomodulators to reduce relapses, there are no treatments as yet that can recover the loss of oligodendrocytes [1-9]. However, the potential adverse effects of immunomodulators remain to be fully studied, especially those affecting the oligodendroglial lineage (demyelinating and/or limiting spontaneous remyelination -see below-). However, recent advances have raised hope in the possible use of neuroregenerative/neuroreparative therapies to complement current treatments.

During embryonic development, oligodendrocyte precursor cells (OPCs) originate in multiple but discrete foci along the neural tube [10-14], thereafter migrating until they reach their final destination where they differentiate into myelin-forming oligodendrocytes. In the mature central nervous system (CNS) of both healthy and sick people, there is a relatively large number of OPCs (around 3-8% of the total cells in the mature brain), which makes this a cell type of interest for cell therapies to repair tissue damage in demyelinating diseases, like MS [3,10,15-19]. Potential therapeutic approaches could include attempts to enhance remyelination by pharmacological manipulation of endogenous OPCs and/or by exogenous OPC engraftment into the injured CNS (cell therapy) [18]. With regards cell therapy, human embryonic stem cell-derived OPCs (hESC-OPCs) or human induced pluripotent stem cell OPCs (hiPSC-OPCs) seem to offer most promise [20-26]. However, experimental data suggests that is not possible to differentiate enough hiPSC derived oligodendrocytes for transplantation, making it more feasible to obtain them from hESCs. However, hESCs are in short supply and they are ethically controversial [27-29]. All these problems may be avoided using endogenous OPCs and by gaining a better understanding of their behavior in order to enhance their remyelination potential, which is the aim of the present study. 

In order to complement and improve the efficiency of pharmacological manipulation of endogenous OPCs as a therapeutic approach in patients with demyelinating diseases, two preliminary steps must be fulfilled. First, it is necessary to define the specific features of the different stages of OPC development, and to improve our knowledge of their proliferation and differentiation properties. As a result, we could then identify key candidates that regulate these changes in pathological brains.

To resolve these questions we have developed an efficient protocol to isolate sufficient OPCs in order to perform biological and pharmacological assays *in vitro* with a view to potentiate their myelination/remyelination potential. We have applied this method to postnatal and adult mouse brains, and to human neurosurgical samples, allowing us to compare the efficiency of our protocol with data obtained previously on newborn mice. In the present work, we present the detailed protocol to obtain OPCs from the postnatal (P15; [30]) and adult (P60 and older; [31]) mouse cerebral cortex, a region with a high density of these cells [32]. The OPCs obtained are viable, and they are capable of migrating and differentiating into myelin forming cells *in vitro*. Moreover, our protocol was more efficient than other methods in terms of the number of OPCs isolated, such as FACS, immunopanning and an Oligodendrocyte Selection Kit (Pesheva WO/2006/067094 A1). Finally, we demonstrate that our protocol is also useful to obtain functional human OPCs from adult brain biopsies [30], which allows us to compare the relative efficiency of our protocol in adult mouse and human brain biopsies. Our protocol opens the door to the performance of further *in vitro* assays to analyze the effects of current MS drugs on endogenous OPCs, as well as for new pharmacological developments to increase the physiological capacities of human OPCs. This protocol has been successfully employed in two published works [30,31] and in several others currently submitted or in preparation.

## Materials and Methods

### Animals

The postnatal (P0, P15) and adult (P60, P180) CD-1 and (P60) C57/BL6 mice used in this study were obtained from Charles River Laboratories and they were maintained in the animal facilities of the Hospital Nacional de Parapléjicos (Toledo, Spain). The animals used at P0 served to compare the data obtained in this study with that available elsewhere. The *plp-GFP* transgenic mouse line [33-35] was also used here because these animals express the *gfp* reporter gene under the control of the *plp* regulatory sequences [34], facilitating the detection of oligodendroglial cells and/or their isolation by FACS [34].

### Human biopsies

Human biopsies of tumor (resection of the safety margins but not the tumor itself) and non-tumor origin (epilepsy, brain traumatism, etc.) were obtained from the Neurosurgery Service at the Hospital La Princesa (Madrid, Spain) and the Neurosurgery Service at the Hospital Virgen de la Salud (Toledo, Spain). All the samples obtained from human brain cortex were from adult patients and the biopsies were transported in an AQIX^®^ RS-I (AQIX Ltd., Imperial College BioIncubator) at 4°C as soon as possible to reduce cell damage. 

### Ethics Statement

All animal experiments were carried out in accordance with Spanish (RD223/88) and European (2010/63/EU) regulations, and they were approved by the Animal Review Board at the “Hospital Nacional de Parapléjicos” (SAPA001). All experiments involving human samples were carried out in accordance with the guidelines of the Research Ethics Committee of Toledo (Spain), which approved our research, and all the subjects provided their written informed consent.

### Materials required for OPC isolation

Flasks were coated for 3-4 h in a tissue culture incubator with poly-L-Ornithine hydrobromide (10 μg/ml, Sigma) diluted in sterile distilled water (~ 7 ml/flask for 75 cm^3^ and 4 ml/flask for 25 cm^3^). After removing the coating solution, the flasks were washed three times with sterile distilled water and dried completely. 

A papain-solution was prepared by diluting papain (0.9 mg/ml, Worthington Biochemical Corp.), L-cysteine (0.2 mg/ml, Sigma) and EDTA (0.2 mg/ml, Roche) in Hanks balanced salt solution without Ca^2+^ and Mg^2+^ (HBSS, Gibco).

The OPC medium used was Dulbecco´s modified Eagle´s media with L-glutamine, 4.5g/L glucose and sodium pyruvate (DMEM, Gibco), supplemented with 10% Fetal Bovine Serum (FBS: BioWhittaker; Lonza) and a 1% Antibiotic Antimycotic Solution (Sigma). 

### New method for postnatal and adult mice OPC isolation

To obtain primary OPC cultures, animals were first sacrificed by cervical dislocation and/or carbon dioxide inhalation (depending on their age), and they were then decapitated with large scissors. The head of the animals was submerged briefly in 70% ethanol to avoid contaminating the dissection with hairs and other material, the skull was rapidly removed in a flow hood after cutting the skin with a scalpel, and the brain was removed with forceps (approximately 5–10 animals could be processed at a time). The brains were placed in a 50 ml conical tube with 10 ml of ice-cold HBSS (with Ca^2+^ and Mg^2+^) and when all the brains had been recovered, the conical tube was warmed for 5 minutes at 37°C in a water bath. A papain-solution was then added and the brains were incubated at 37°C in the water bath. Both the optimal concentration of the papain-solution and the length of incubation at 37°C depend on the age of the animals (Table 1). This step was crucial to facilitate the elimination of the meninges, which are more strongly attached to the parenchyma at adult stages than in neonatal animals. The papain reaction was stopped by adding 20 ml of HBSS (with Ca^2+^ and Mg^2+^) to the conical tube and placing it on ice. Then brains were then removed one by one, placed in HBSS (with Ca^2+^ and Mg^2+^) in a glass Petri dish, and the remaining meninges and as much of the choroid plexus as possible were removed with forceps under a dissection microscope as fast as possible to avoid cell death. Once the meninges and choroid plexus had been removed, the olfactory bulbs and the cerebellum were also eliminated with the forceps, and the brain was split into two along the sagittal midline axis, also removing the diencephalon. 

**Table 1:** Summary of the details and the protocol’s relative efficiency.

	**Animals/ flask**	**Meninges removal**	**Tissue digestion**	**Number of OPCs/animal**	**OPCs %relative to P0**	**% OPCs/total glial cells in cerebral cortex**
**P0**	3	PS (1:10) in HBSS, 5 min	PS (1:10) in HBSS, 5 min	263,148±6,508	100%	12.9%
**P15**	5	PS (1:5) in HBSS, 5 min	PS (1:10) in HBSS, 10 min	123,194±8,774	50%	6.3%
**P60**	5	PS (1:5) in HBSS, 10 min	PS (1:10) in HBSS, 15 min	68,444±5,461	25%	3.8%
**P180**	5	PS (1:5) in HBSS, 10 min	PS (1:10) in HBSS, 15 min	39,757±4,410	12%	2.02%

The enzyme dilution and the digestion time for the meninges/choroid plexus and the cerebral cortex depend on the postnatal stage analyzed. The number of OPCs isolated was also stage-dependent and decreased with age. In addition, we indicate the %OPCs with respect to the cerebral cortex glial cells in the primary cultures. PS: Papain-solution

The brain cortices were then placed in a clean petri dish in 10 ml of ice-cold HBSS (without Ca^2+^ and Mg^2+^). The absence of these cations favors papain digestion. The tissue was then dissociated mechanically, first with small dissecting scissors and then by transferring the content of the petri dish into a 50 ml conical tube, warming it to 37 °C for 5 minutes and pipetting the tissue through a serological plastic pipette. Small pieces of tissue (approx. 2 mm^3^) were incubated with different dilutions of papain-solution for distinct times in function of the age of animal (Table 1). After enzymatic digestion, up to 30 ml (final volume) of OPC medium and 300 μl of a DNAse solution (1%, Sigma) were added to stop the papain digestion. The tissue suspension was then triturated through a pipette several times, incubated at 37°C for 5 minutes and centrifuged at 900 rpm for 10 minutes. The supernatant and the most superficial portion of pellet (myelin debris) was removed and the remaining pellet was resuspended in 10 ml of OPC medium (first with a serological plastic pipette and then with an eppendorf p1000 pipette). The cell suspension was passed through a 100 µm nylon mesh strainer (BD Biosciences) and the flow-through was collected in a 50 ml conical tube. Finally, OPC medium was added in function of the final number of flasks, and the cells recovered were seeded in 75 cm^2^ Poly-L-Ornithine coated flasks in a final volume of 10 ml per flask. The cells were incubated at 5% CO_2_ and 37°C (the ratio animals/flask depends on the age of the animal: Table 1), and after one day, the OPC medium was changed to avoid cell death, replacing the OPC medium with OPC medium supplemented with PDGF-AA (10ng/ml, Millipore). For the differentiation assays, the cultures should not be supplemented with PDGF-AA for more than 10-15 days, at which time the supplemented medium was again replaced with OPC medium. In the case of P0 cultures, supplementation with PDGF-AA was unnecessary. The medium in the flasks was changed every 2-3 days during culture. After 15-20 days in culture for P15 brain cortices, or 25-35 days for P60 and P180 cortices, a monolayer mixture of astrocytes, microglia and OPCs should be evident. At this point the culture was ready to be shaken to obtain purified OPCs and the proportion of isolated OPCs with respect to the total number of glial cells in the primary cultures was assessed (Table 1).

To purify OPCs, the culture flasks were removed from the incubator, the flask caps were screwed on tightly and the flasks were shaken overnight (18–20 h) in an orbital shaker at 250 rpm and 37 °C. Subsequently, the medium containing the detached OPCs was collected rapidly and passed through a 40 μm nylon mesh strainer placed over a 50 ml conical tube. The flow-through was collected and centrifuged at 900 rpm, for 10 minutes, and the pellet recovered was then resuspended in 10 ml of OPC medium. These cells were then plated on an untreated plastic Petri dish at 37°C and left for 45 minutes, allowing the microglial cells to attach but not the OPCs. The unattached cells were collected and the process was repeated again for 30 minutes, after which the OPC-enriched supernatant was collected and centrifuged at 900 rpm for 10 minutes. The supernatant was discarded, and the pellet containing the purified OPCs was resuspended in OPC medium, counting the live cells using the Trypan blue exclusion assay. Finally, the OPCs purified were plated in the corresponding medium and at the concentration required by the assay (see below), and they were incubated at 37°C in 5% CO_2_.

### Modifications to isolate OPCs from human brain biopsies

The protocol described above was used to isolate OPCs from human brain biopsies with minor modifications. Rather than flasks of 75 cm^2^, 25 cm^2^ flasks were used with a final volume of 5 ml OPC medium per flask, as opposed to 10 ml. While this protocol worked with at least 0.5 grams of fresh tissue, the best efficiency was obtained with 4 grams fresh weight. Enzymatic digestion of the meninges and choroid plexus was performed in the papain solution at a 1:2.5 dilution for 10 minutes and the remaining tissue was digested at 1:10 for 15 minutes. Cultures reached confluency after 30-40 days and the flasks were shaken at 230 rpm instead of 250 rpm.

### Immunocytochemistry for OPC identification and image acquisition

**Figure 1:**
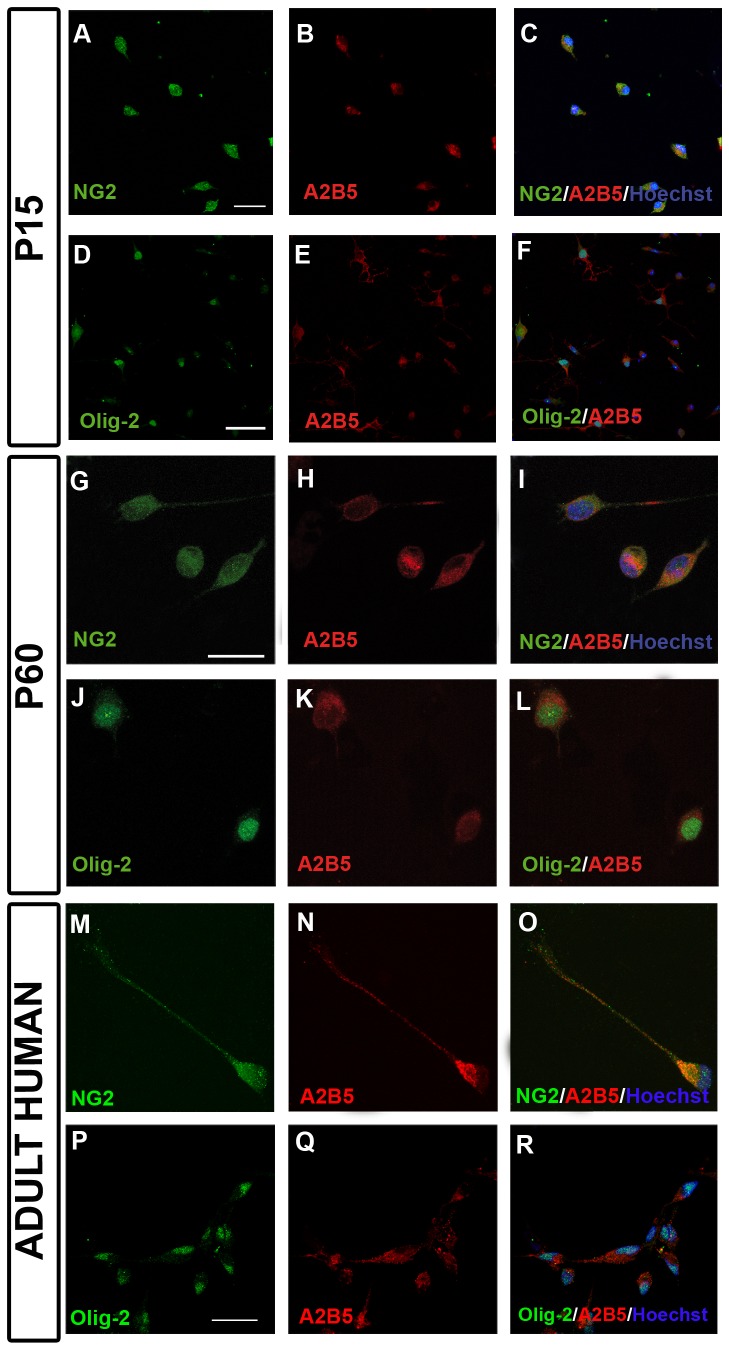
Identification of OPCs. (**A**-**F**) OPCs derived from p15 CD-1 mice after 24 hours in culture. OPCs were identified by dual immunostaining for NG2^+^/A2B5^+^ (**A**-**C**) and Olig-2^+^/A2B5^+^ (**D**-**F**). (**G**-**L**) OPCs derived from p60 CD-1 mice after 24 hours in culture. OPCs were dual-labeled with NG2^+^/A2B5^+^ (**G**-**I**) and Olig-2^+^/A2B5^+^ (**J**-**L**). (**M**-**R**) Identification of OPCs derived from human biopsies after 24 hours in culture. Images show the co-expression of NG2/A2B5 (**M**-**O**) and Olig-2/A2B5 (**P**-**R**). Scale bar represents 25 µm in A-F or 10 µm in G-R.

The identification of OPCs (Figure 1) was carried out after 24 hours in culture by dual fluorescence immunocytochemistry using the A2B5 (diluted 1:10, Hybridoma Bank) and NG2 (1:200 diluted, a gift from Prof. William Stallcup) cytoplasmic antibodies, and the nuclear Olig2 antibody (diluted 1:200, Millipore). To identify differentiated oligodendrocytes, dual immunocytochemistry was carried out with the anti-CNPase (1:200, Covance) and Olig2 antibodies. Images were obtained on a SP5 resonant scanning confocal microscope (Leica Microsystems).

### Comparison with other methods

To address the efficiency of our OPC isolation protocol we developed and characterized reproducible primary cultures of cortical derived OPCs using two existing methods.

Firstly we used Fluorescence Activated Cell Sorting (FACS) to isolate OPCs from the cerebral cortices of 5 plp-GFP transgenic mice (P15, P60) that were dissociated with the Neural Tissue Dissociation Kit T (Miltenyi Biotec; [36,37], according to the manufacturer’s instructions. The dissociated cells were filtered through a 40μm cell strainer and centrifuged at 900 rpm to increase the OPC purity for the subsequent FACS isolation. We then resuspended the cell pellet in 10 ml of ice-cold 0.9 M sucrose solution diluted in HBSS and we immunostained the cells with anti-A2B5 antibodies conjugated to phycoerythrin (Miltenyi Biotec), following the manufacturer’s instructions. Cells co-expressing A2B5 and GFP were sorted on a FACS Aria TM flow cytometer (BD Biosciences) in HBSS buffer with 2% FBS, 25 mM HEPES buffer solution (Fluka Biochemika) and 5 mM EDTA (Roche). The OPCs were collected in HBSS buffer and the live cells were counted using the Trypan blue exclusion assay. OPCs were plated at the desired density (see above) and incubated at 37°C in 5% CO_2_.

Secondly, we used an Oligodendrocyte selection Kit (Pesheva WO/2006/067094 A1) according to the manufacturer’s instructions.

### Assays carried out on isolated OPCs

Once purified OPCs had been obtained, we could perform different assays to study their behavior. OPCs were plated in the corresponding medium at the appropriate concentration for the assays performed. 

For survival and differentiation assays [30], cells were seeded (2x10^4^ cells/coverslip) on Poly-L-lysine (Sigma, 0.1 mg/ml in borate buffer) and laminin (Sigma, 10 μg/ml in 1X sterile PBS) coated coverslips. Differentiation medium was used in these assays [38], containing: BME:F12 (1:1, Gibco) supplemented with 100 μg/ml of holo-transferrin (Sigma), 20 μg/ml of putrescine (Sigma), 12.8 ng/ml of progesterone (Sigma), 10.4 ng/ml of sodium selenite (Sigma), 25 μg/ml of insulin (Sigma), 0.8 μg/ml of thyroxine (Sigma), 0.6% D(+)-glucose (Normapur), 6.6 mM L-glutamine (Gibco) and 1% Antibiotic Antimycotic Solution (Sigma). First, drops of medium (50 μl) containing purified OPCs were seeded onto the coated coverslips situated in multiwell plates and they were allowed to attach for 1 hour before 450 μl of the corresponding medium was added. The cultures were then maintained in an atmosphere of 5% CO_2_ at 37°C for several days depending of the origin of OPCs [30]. 

As previously reported [33,39,40], for chemotaxis and proliferation assays the cells were seeded in Bottenstein and Sato defined medium [41] consisting of DMEM with 1% FBS, 1% L-glutamine (Gibco), 0.03% BSA (Sigma), 1% antibiotic/antimycotic Solution (Sigma), 0.3 ng/ml 3,3´,5-triiodo-L-thyronine (T3, Sigma), 0.4 ng/ml thyroxine (Sigma), 16 µg/ml putrescine (Sigma), 40 ng/ml sodium selenite (Sigma), 9.3 µg/ml insulin (Sigma), 0.1 mg/ml holo-transferrin (Sigma) and 62 ng/ml progesterone (Sigma).

### Statistical Analysis

The data is shown as the mean ± SEM and it was analyzed using SigmaPlot software (Jandel Scientific). A comparative analysis was performed using a Student´s t-test (or Mann-Whitney rank sum test). Statistical significance was set at P<0.05: * P<0.05, ** P<0.01, *** P<0.001.

## Results

When compared to their isolation from neonatal mice, one of the principal problems to be addressed when isolating OPCs from adult CNS tissue is the efficiency with which they are obtained. However, with the protocol described here we were able to obtain enough OPCs to perform different assays and to study their biology.

### Isolated OPCs from adult mice and human expressed the same molecular markers as their embryonic and early postnatal counterparts

OPCs isolated from the postnatal mouse cortex (P15, Figure 1 A-F and P60, Figure 1 G-L) using our protocol mostly showed the typical bipolar morphology after 1 day in culture (Figure 1). At the three stages analyzed (P0, P15, P60), OPCs were double labeled with A2B5/NG2 antibodies and with A2B5/Olig2 antibodies, indicating that the vast majority of the cells isolated were postnatal OPCs that expressed early markers of this cell type (Figure 1 A-L). Like murine OPCs, OPCs isolated from human biopsy tissue also expressed early OPCs markers (Figure 1 M-R).

### Strain- and stage-dependent differences in the number of OPCs obtained

**Figure 2:**
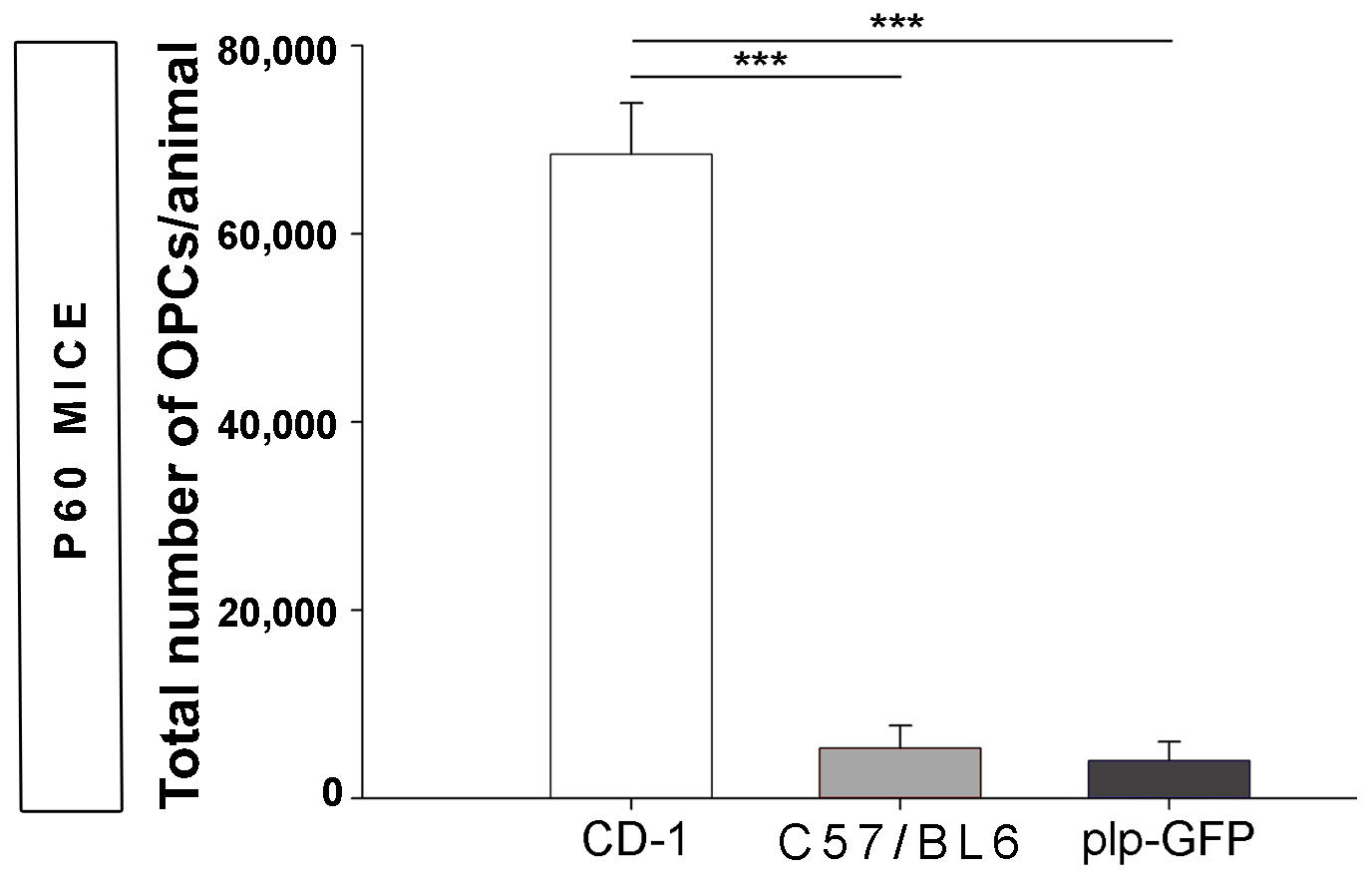
Strain-dependent differences in the OPCs isolated from the P60 mouse cortex. More OPCs were obtained from CD-1 mice than from C57/BL6 or *plp*-GFP mice (C57/BL6 background). Values are the mean ± SEM and they were analyzed with a *t*-test: *P<0.05, **P<0.01 and ***P<0.001.

To determine the efficiency of our protocol, we assessed the number of OPCs isolated from different mouse strains. A significantly different number of adult (P60) OPCs was obtained in the strains analyzed (Figure 2), with the highest number of adult OPCs obtained from CD-1 mice and significantly fewer OPCs isolated from the C57/BL6 strain and *plp*-GFP transgenic mice (bred on a C57/BL6 genetic background). In the light of these results, we decide to employ CD-1 mice for further experiments.

We then assessed the efficiency of the procedure in CD-1 wild type mice in function of the age of the tissue used (Table 1). The largest number of OPCs was isolated from P0 brains and from this stage onwards, the number of OPCs decreased gradually from P15 to P180. Considering the total number of OPCs at P0 stage as the reference value (100%), the percentage of OPCs obtained at postnatal and adult stages were approximately 50% (P15), 25% (P60) and 12% (P180: Table 1). The number of OPCs was also expressed with respect to the glial cells also isolated in primary cultures from the cerebral cortex (Table 1) and a similar trend was observed.

### This new protocol was more efficient than other methods to isolate adult OPCs

**Figure 3:**
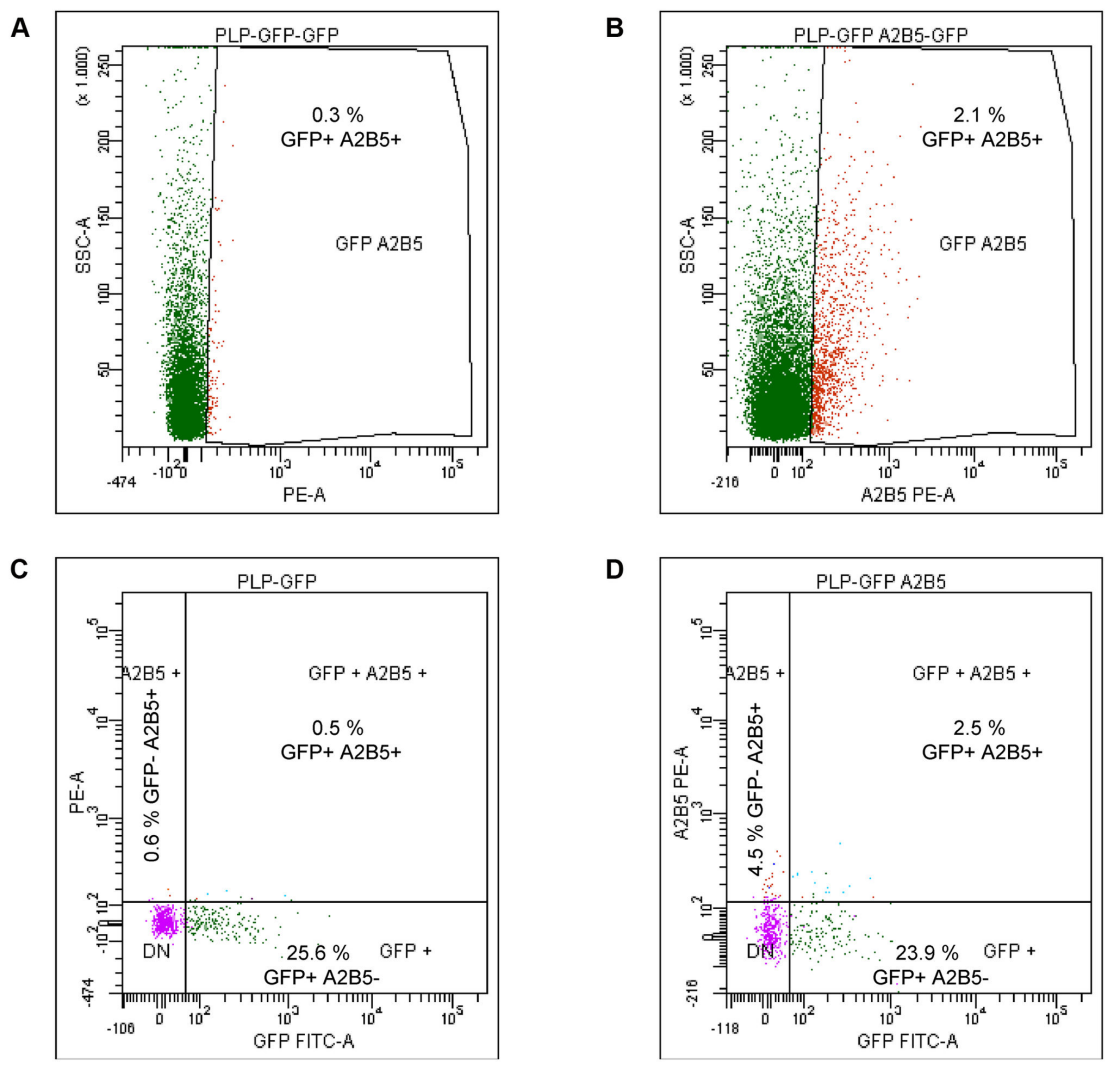
FACS sorting of adult OPCs from transgenic mice. Histograms of unstained cells (**A**) and those stained with an anti-A2B5-PE antibody (**B**). Histograms showing the relative percentage of the different cell populations (GFP^-^/A2B5^+^, GFP^+^/A2B5^-^, GFP^+^/A2B5^+^) in experiments with no labeling (**C**) and with anti-A2B5-PE staining (**D**).

To corroborate the efficiency of our new protocol, we compared our results with other well-known techniques. Firstly, we isolated OPCs from both postnatal (P15) and adult mice (P60) using FACS technology [36,37], taking advance of the *plp*-*GFP* transgenic mice. Using FACS, we were able to sort OPCs defined by the co-expression of GFP and A2B5. Consistent with our earlier results (see above), the number of OPCs decreased with age: at P15 around 20% cells co-expressed GFP and A2B5, while at P60 only around 2% of cells displayed this molecular phenotype (Figure 3). However, a large amount of cell death occurs during the sorting process because less than 50% of the total cells sorted were alive on the day after performing FACS. In adult specimens, and despite using enzymatic digestion to reduce the processing time *ex vivo* and the cell death, the total number of isolated OPCs from cerebral cortices per animal was only around 10,000, which is <15% the number obtained with our protocol (Table 2). 

**Table 2:** Comparison of the methods available to isolate OPCs with respect to our new protocol.

**Stage**	**CNS region**	**Method**	**Number of OPCs/animal**	**Relative efficiency of our present protocol**
**P0**	Forebrain	Oligodendrocyte Selection Kit	250,000	Similar
	Cerebral cortex	MACS [48]	368,000	1.4-fold lower
**P15**	-	-	-	-
**P60**	Optic nerve (*)	Immunopanning [49]	2,000-2,500	30-fold higher
	Cerebral cortex	Oligodendrocyte Selection Kit	7,500	10-fold higher
	Cerebral cortex	FACS	10,000	7-8-fold higher
**P180**	-	-	-	-

(*) Data from the rat.

When OPCs were isolated using the Oligodendrocyte Selection Kit (Pesheva WO/2006/067094 A1), the number of OPCs purified from adult mice (P60) was clearly lower than those obtained with our novel protocol. Despite introducing enzymatic digestion of the meninges and choroid plexus to diminish cell death, the number of live OPCs recovered by this procedure was only 7,500/animal, which is approximately 10% the number obtained with our original protocol (Table 2).

### A large number of functional OPCs were obtained from biopsies of adult human brain cortex

**Figure 4:**
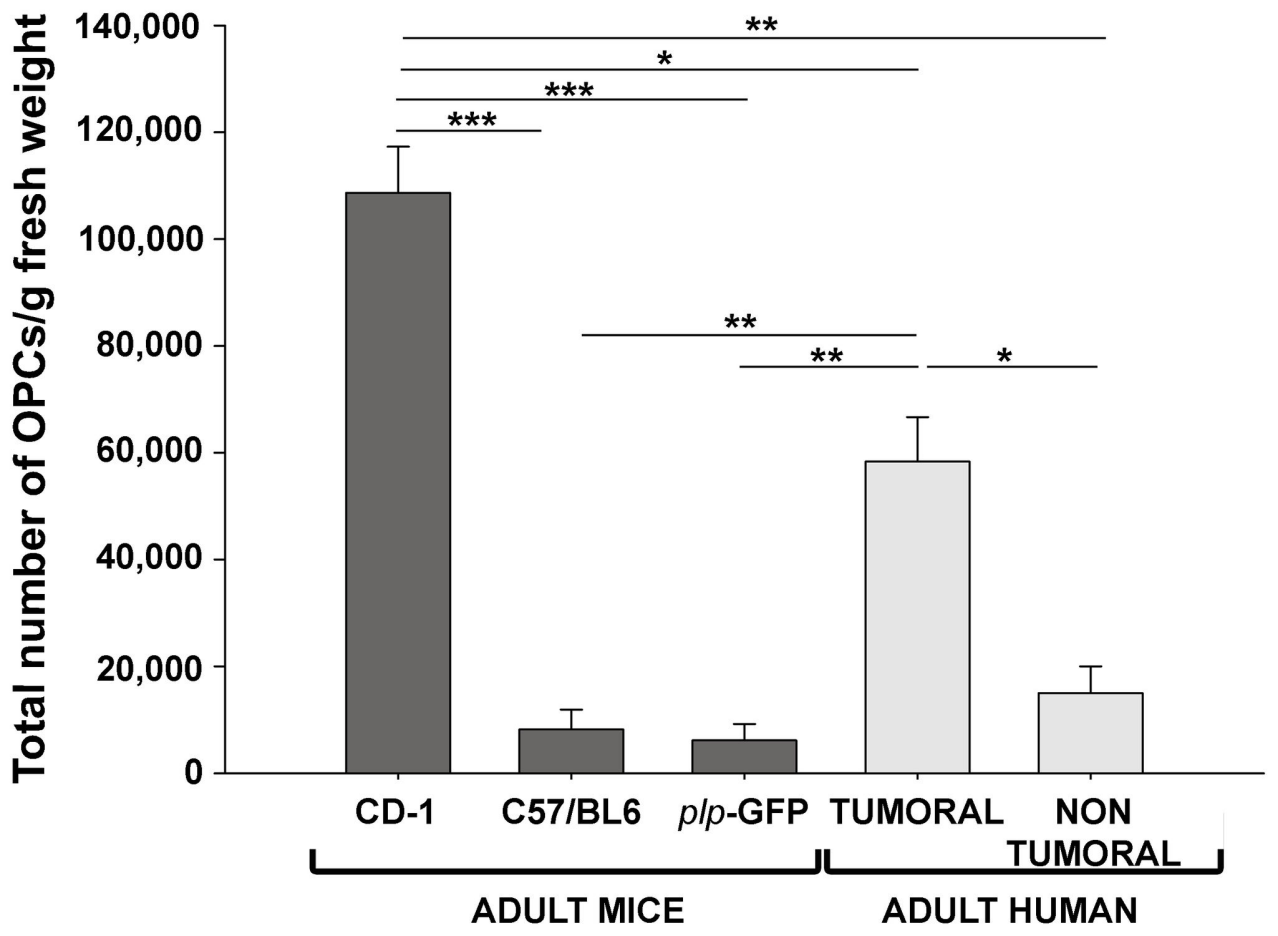
Protocol efficiency. Comparison of the efficiency of the protocol in obtaining OPCs from the adult mouse cerebral cortex (CD-1, C57/BL6 or *plp*-GFP, P60) and from adult human biopsies (resection margins of tumors and non-tumor tissue). The amount of OPCs/gram fresh weight from both types of human biopsy (non tumor and tumor) was higher than that from the brain cortex from C57/BL6 and *plp*-GFP adult mice. The values are given as the mean ± SEM and they were analyzed with a *t*-test: *P<0.05, **P<0.01 and ***P<0.001.

When we compared the efficiency of the isolation protocol in terms of the % OPCs/gram fresh weight of the starting material, significant differences were observed between the different tissues used: mouse cerebral cortex, resection margins of human brain tumors, non-tumor human brain tissue (Figure 4). With our protocol we obtained approximately 100,000 OPCs/gram fresh weight from adult CD-1 mice, 50,000 OPCs/gram fresh weight from the resection margins of human brain tumors, and 15,000 OPCs/gram fresh weight from non-tumor human brain tissue (Figure 4). Significantly, we obtained more OPCs/gram fresh weight from both types of human biopsies (non-tumor and tumor) than from the cortex of the two other strains of adult mice employed in this study: C57/BL6 (around 10,000) and *plp*-GFP (around 5,000: Figure 4).

### OPCs isolated from the different samples were functional and useful for further biological assays

**Figure 5:**
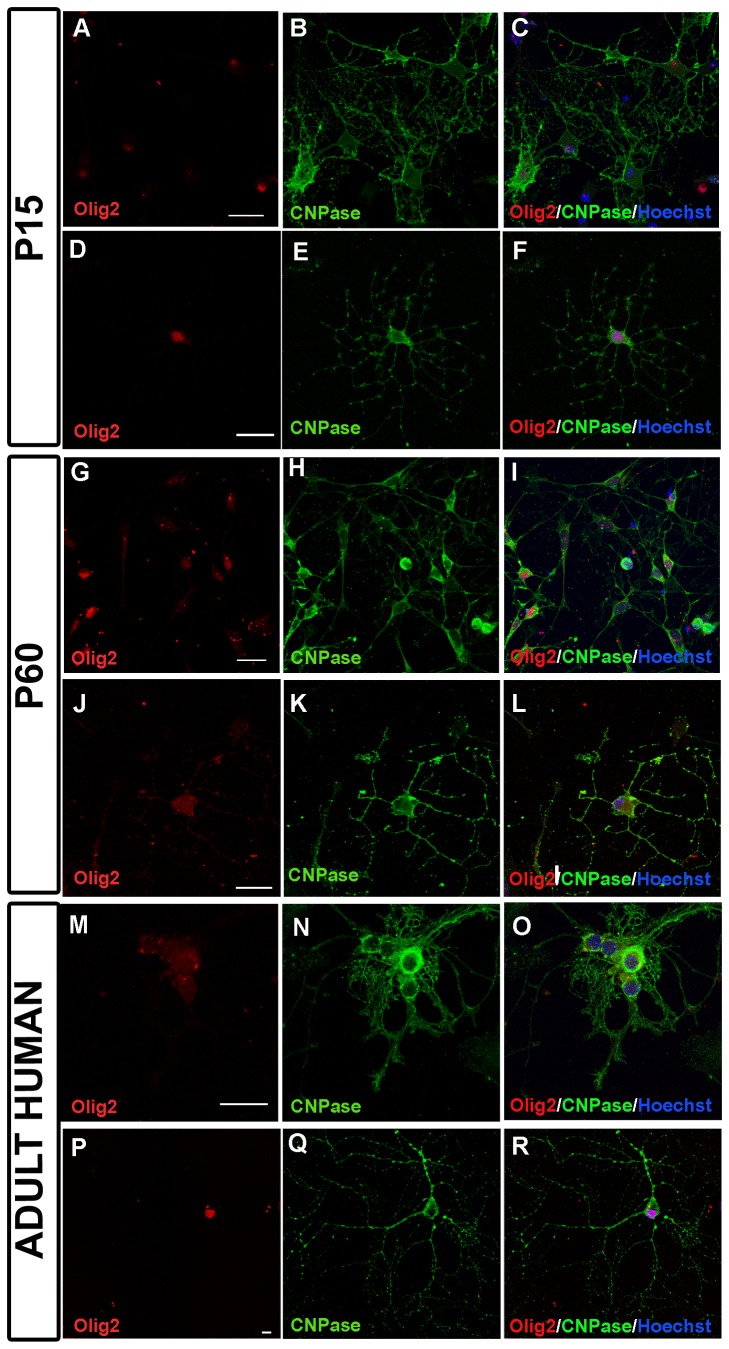
Identification of oligodendrocytes. (**A**-**F**) Low (**A**-**C**) and high (**D**-**F**) magnification images showing cells from P15 CD-1 mice expressing Olig2 and CNPase, identified as oligodendrocytes after 7 days in culture. (**G**-**L**) Low (**G**-**I**) and high (**J**-**L**) magnification images of cells from P60 CD-1 mice expressing Olig2 and CNPase, identified as oligodendrocyte after 7 days in culture. (**M**-**R**) Images of cells from human biopsies expressing Olig2 and CNPase, identified as oligodendrocytes after 5 days in culture at low (**M**-**O**) and high (**P**-**R**) magnification. Scale bar: 25 µm.

Differentiation assays demonstrated that at all the stages studied, the OPCs isolated from the mouse cortex with our new procedure were viable and could differentiate into mature oligodendrocytes (Figure 5). OPCs isolated from adult mice were cultured for 7 days *in vitro* in differentiation medium (see methods) and they differentiated into mature, CNPase-Olig2 positive oligodendrocytes, as were also detected when P15 (Figure 5 A-F) and P60 (Figure 5 G-L) tissue was used. In the case of human derived OPCs cultures, CNPase-Olig2 positive cells could also be detected after 5 days *in vitro* in differentiation medium (Figure 5 M-R).

The protocol that we describe here has already been used in our laboratory and the OPCs isolated in this way have been shown to be functional and useful for assays of migration [31], proliferation, survival and differentiation [30].

## Discussion

We describe here a protocol that we have established to obtain OPCs from the mouse cerebral cortex during maturation and adulthood, as well as from human brain biopsies, fulfilling an important gap in the field. The main difference with other similar protocols is that we have incorporated a step to perform a controlled enzymatic digestion of the meninges and choroid plexus (Table 1), which dramatically decrease the time the tissue spends *ex vivo*, thereby reducing the cell death in mature/adult mouse and human tissues.

As a preliminary study, we compared the total number of OPCs obtained from different mouse strains (CD-1, C57/BL6, *plp*-*GFP*) as strain-dependent differences have been reported for cortical demyelination in the murine cuprizone model [42], microglial-macrophage synthesis of tumor necrosis factor after focal cerebral ischemia [43], protection against Theiler's murine encephalomyelitis virus (TMEV; [44]), axonal regeneration [45] and adult hippocampal neurogenesis [46]. Indeed, it was recently shown that there are also strain-specific differences in perinatal rodent oligodendrocyte lineage progression and its correlation with that in humans [47]. Our results demonstrate that the number of OPCs isolated from adult mice cerebral cortices is strain-dependent, with the highest number of OPCs isolated from the CD-1 strain. 

To compare this protocol with current standard procedures to isolate early postnatal OPCs, we used the cerebral cortex of P0 mice, particularly since the only data about the number of OPCs purified from species has been obtained from the early postnatal forebrain (Oligodendrocyte Selection Kit, Pesheva WO/2006/067094 A1) or cerebral cortex [48], although some data from the adult rat optic nerve is also available (immunopanning: Barres WO/1997/007200; [49]). The Oligodendrocyte Selection Kit employs a protocol that is based on the selective adhesion of oligodendrocytes to Tenascin-R coated onto special petri dishes under patent (Pesheva WO/2006/067094 A1), and it has been established that using this kit around 10^6^ OPCs can be obtained from the whole forebrains of four P2 mice. Thus, the number of OPCs obtained from early postnatal mice forebrains (around 250,000/animal) is similar to that obtained with our protocol. However, when the Oligodendrocyte Selection Kit was used to isolate OPCs from the adult (P60) cerebral cortex it was significantly less efficient than our protocol. Indeed, approximately 10-fold more adult OPCs were obtained with our protocol, representing an additional advantage in terms of the number of animals that must be sacrificed, a benefit that is consistent with the Guidelines for the Care and Use of Mammals in Neuroscience and Behavioral Research (2003) of the National Research Council, as well as representing a saving in terms of time and costs. The reduction in the adult OPCs obtained with the Kit could reflect changes in the expression of tenascin-R in adult animals with respect to that at perinatal stages. 

Isolation by magnetic cell sorting (MACS; [48]), is not much more efficient than our protocol (3.68 × 10^5^ ± 9.17 × 10^4^ cortical OPCs/brain), and it can only be used to isolate OPCs from P0-P7 mice and not from adult mouse or human tissue. FACS would appear to be a viable alternative in mice, as it allows small subpopulations of *in vivo* fluorescent cells to be isolated from transgenic animals with excellent specificity [50]. However, in our experiments using *plp*-*GFP* transgenic mice, fewer OPCs co-expressing both cell surface molecular markers were obtained with this technique, limiting the use of the isolated cells for further studies. One putative explanation may be that during the pre-sorting and sorting steps, cells are incubated for several hours in a non-natural microenvironment, and they are subjected to enzymatic digestion, factors that are associated with cell stress and cell death, as seen previously for embryonic radial glia (69±15% live cells; [51]) and embryonic stem cells (30% live cells; [52]). In summary, our present protocol was around 7-fold more efficient than using FACS.

Immunopanning is an expensive technique [53-55] that is widely used to isolate OPCs from the rat CNS (Barres WO/1997/007200; [49,56-61]), however there are currently no antibodies suitable to select OPCs from mice with this technique [62]. Nevertheless, a new immunopanning approach using rat anti-mouse PDGFRα following a negative selection with BSL-I has recently resolved this problem, at least for the optic nerve [61]. A second approach that has been established to purify OPCs from young mouse brains (P13) involves three-step immunopanning using mouse anti-mouse Thy1.2, anti-mouse GC and anti-mouse O4 antibodies [61]. However, as far as we know, there are no data available regarding the number of OPCs isolated from the adult mouse cerebral cortex to compare the relative efficiency of this immunopanning protocol with that of ours. The only data available regarding immunopanning efficiency comes from the adult rat optic nerve in which a many fewer OPCs can be isolated: a mere 1,000-1,250 OPCs per optic nerve (Barres WO/1997/007200; [49]). As occurs with the Oligodendrocyte Selection Kit, the 30-fold increase of the efficiency with our protocol can be associated with a reduction in the number of experimental animals that must be sacrificed.

There is no information available regarding the efficiency of protocols to purify cortical OPCs from adult humans in order to compare such data with our own results. The percentage of OPCs obtained in the only study published to date on the isolation of cortical OPCs indicates that only 1% of the cells obtained were A2B5^+^ cells, and that the majority of the cells obtained were identified as pre-oligodendrocytes or mature oligodendrocytes [63]. In addition, the previous protocols to isolate A2B5^+^ cells, which labels neurons, astrocytes and oligodendrocytes in the human CNS [64,65], obtained those cells from sub-cortical white matter [66,67] with an isolation efficiency of 84% mature oligodendrocytes and only 16% OPCs [66], or without specifying the CNS region [68]. Thus, as far as we know, our protocol is the first to obtain OPCs from human adult brain biopsies as well as from adult mice. The capacity to isolate OPCs from these tissues allows us to directly compare data from adult mice with that from humans, which is extremely useful given the difficulties in obtaining adult human brain biopsies. 

In the case of human OPCs, hESC-OPCs or hiPSC-OPCs appear to be alternative sources of material for cell therapies. Indeed, these cells represent a promising therapeutic option [20-26] because they have been shown to be capable of restoring locomotor function and they can increase myelination in rats with spinal cord injury [22], as well as restoring myelination in the hypomyelinated shiverer mouse brain [21,23,25,26]. However, in preclinical trials using hESC-OPCs (GRNOPC1), non-proliferative epithelial cysts occasionally appeared that were confined to the injury site (Geron Corp.; [22]). Moreover, experimental data suggests that the differentiation of sufficient numbers of oligodendrocytes for transplantation from hiPSCs or hESCs may have to address technical and ethical problems [27]. Moreover, the use of this kind of cell may lead to the formation of tumors, while the transplantation of more differentiated cells will not necessarily produce a pool of stem cells with the capacity to continually supply newly differentiating cells if the transplanted cells die. Such cell-based therapeutic agents for use in humans must also be free of animal contaminants that may contain pathogens or elicit an immune reaction after transfer to a host. Also this type of cell transplant may also be subject to immune rejection [28,29]. Such problems could be avoided by using endogenous OPCs that could be isolated using the protocol presented here.

In conclusion, we present here a “basic protocol” to obtain adult OPCs from humans and mice. For the first time, our protocol opens the way to obtain sufficient quantities of adult murine and human OPCs to permit the effects of current treatments for MS, such as Natalizumab, Fingolimod or other immunomodulators, on OPC neurobiology to be analyzed (survival, proliferation, migration, differentiation) [30,31], as well as the effects of new molecules that favor remyelination together with the immunosuppresion. Moreover, this protocol will facilitate the search for alternative therapeutic treatments for demyelinating diseases with less adverse side effects, advancing their testing in clinical trials. Finally, improving the protocol to isolate murine and human adult OPCs will help advance our knowledge about the use of these cells in cell therapy. Information regarding cell therapy can be obtained from murine models that mimic MS, such as experimental autoimmune encephalomyelitis (EAE) and/or TMEV, avoiding the ethical implications of using hESCs or hiPSCs [28]. Thus, our protocol will undoubtedly contribute to advance our understanding of the molecular and cellular mechanisms involved in demyelinating diseases.
